# 

**Published:** 1898-11

**Authors:** 


Fifty-Fourth Year.

VOL. LIV.
WHOLE NUMBER,
DCXXIV.

NOVEMBER, 1898.

New Series.
VOL. XXXVIII.
No. 4.

LIBRARY
Indexed
S. G. O.

### BUFFALO MEDICAL JOURNAL

Established 1845 by AUSTIN FLINT, M. D.

EDITORS:

THOS. LOTHROP, M. D. WM. WARREN POTTER, M. D.

ASSOCIATE EDITORS:

WILLIAM C. KRAUSS, M. D.
JOHN A. MILLER, PH. D.
ERNEST WENDE, M. D.
JAMES WRIGHT PUTNAM, M. D.
JOHN PARMENTER, M. D.
ALVIN A. HUBBELL, M. D.

LIBRARY
SURGEON GENERAL'S OFFICE
NOV.-3--1898

SCRIPTA FERUNT ANNOS

EUROPEAN AGENCY, J. B. BAILLIÉRE & FILS, 19 Rue Hautefeuille, Paris.
*Subscription, if paid in advance, $2.00; otherwise, $2.50. Foreign subscription, $2.50.*
Copyright 1898, by WILLIAM WARREN POTTER, M. D.
Entered at the Post Office at Buffalo, N. Y., as second-class mail matter.

A. T. BROWN PRINTING HOUSE, BUFFALO, N. Y.

Table of Contents on Page V.

